# Expanding the Microcolonial Black Fungi *Aeminiaceae* Family: *Saxispiralis lemnorum* gen. *et* sp. nov. (*Mycosphaerellales*), Isolated from Deteriorated Limestone in the Lemos Pantheon, Portugal

**DOI:** 10.3390/jof9090916

**Published:** 2023-09-10

**Authors:** Diana S. Paiva, João Trovão, Luís Fernandes, Nuno Mesquita, Igor Tiago, António Portugal

**Affiliations:** 1 Centre for Functional Ecology (CFE)—Science for People & the Planet, Department of Life Sciences, University of Coimbra, Calçada Martim de Freitas, 3000-456 Coimbra, Portugal; 2 FitoLab—Laboratory for Phytopathology, Instituto Pedro Nunes (IPN), Rua Pedro Nunes, 3030-199 Coimbra, Portugal; 3 TERRA—Associate Laboratory for Sustainable Land Use and Ecosystem Services, Department of Life Sciences, University of Coimbra, Calçada Martim de Freitas, 3000-456 Coimbra, Portugal

**Keywords:** Ançã limestone, biodeterioration, cultural heritage, new fungal species, fungal phylogeny, rock-inhabiting fungi, fungal taxonomy

## Abstract

With an impressive ability to survive in harsh environments, black fungi are an ecological group of melanized fungi that are widely recognized as a major contributor to the biodeterioration of stone cultural heritage materials. As part of the ongoing efforts to study the fungal diversity thriving in a deteriorated limestone funerary art piece at the Lemos Pantheon, a national monument located in Águeda, Portugal, two isolates of an unknown microcolonial black fungus were retrieved. These isolates were thoroughly studied through a comprehensive analysis based on a multi-locus phylogeny of a combined dataset of ITS rDNA, LSU, and *rpb2*, along with morphological, physiological, and ecological characteristics. Based on the data obtained from this integrative analysis, we propose a new genus, *Saxispiralis* gen. nov., and a new species, *Saxispiralis lemnorum* sp. nov., in the recently described *Aeminiaceae* family (order *Mycosphaerellales*). Prior to this discovery, this family only had one known genus and species, *Aeminium ludgeri*, also isolated from deteriorated limestone. Additionally, considering the isolation source of the fungus and to better understand its potential contribution to the overall stone monument biodeterioration, its *in vitro* biodeteriorative potential was also evaluated. This work represents a significant contribution to the understanding of the fungal diversity involved in the biodeterioration of limestone heritage.

## 1. Introduction

Our understanding of rocks as inert substrates devoid of life has been challenged over time with the discovery of lithobiontic organisms [1,2]. Lichens, cyanobacteria, chemoorganotrophic bacteria, and fungi are among the dwellers commonly found within these rock micro-communities [3]. These organisms inhabit the surface and interior of rocks (epi- and endolithic, respectively) and engage in interactions with the rock matrix, playing an active role in the alteration and transformation of rock substrates, which carry important ecological and biogeochemical consequences [4,5,6,7,8].

Fungi are one of the most ancient and diverse groups of organisms that can be found worldwide, successfully colonizing and flourishing in every biome, including extreme environments as far apart as the glacial valleys of Antarctica [9,10,11,12] and the hot and arid deserts [13,14], as well as habitats resulting from human activity such as acid mine waters, sewage and industrial effluents [15], areas contaminated with nuclear radiation [16], salterns [17], and many others. With their remarkable versatility and resilience, fungi can adopt various structural, morphological, and metabolic strategies to ensure their growth and survival on multiple substrates [18]. They are widely recognized as one of the most important stone colonizers [19,20], thriving in both natural and manmade rocky materials, and playing a pivotal role in the deterioration of these substrates, including stone cultural heritage artifacts, such as sculptures, monuments, and relics, causing aesthetic, chemical, physical, and mechanical alterations [19,20,21,22,23,24,25,26], ultimately resulting in economic, artistic, and historic losses.

Among fungal biodeteriogens, a specialized yet diverse group of melanized fungi are amidst the persistent settlers of rocks. Commonly known as black fungi (BF), among other names such as rock-inhabiting fungi (RIF) or microcolonial fungi (MCF) [27], these organisms represent a heterogeneous taxonomic group, encompassing several orders within the classes *Dothideomycetes* (e.g., order *Mycosphaerellales*) and *Eurotiomycetes* (e.g., order *Chaetothyriales*) in the phylum *Ascomycota* [28,29,30,31,32,33,34]. Despite their phylogenetic diversity, these fungi form an ecological group specialized in extremotolerance, exhibiting a set of convergent features that reflect their adaptation to harsh and extreme environments [34]. These morphophysiological traits related to stress tolerance include phenotypic plasticity, the ability to transition from a mycelial to a meristematic state, microcolonial growth, simple life cycles, and dispersal mechanisms reliant on vegetative fragmentation or poorly differentiated conidia-like cells [10,17,35,36,37,38,39,40]. Among these features, the most prevalent and distinctive is the presence of strongly melanized cell walls, with melanin being a major stress-protective compound/pigment [39]. Additionally, these fungi produce other protective molecules such as mycosporines and carotenoids [41,42]. They are a poikilotolerant group, with these remarkable adaptations enabling them to tolerate a wide range of stress factors, including extreme temperatures, high solar and UV radiation, oligotrophy, limited water availability, and variations in salinity and pH, commonly encountered on rock surfaces [35,36,37,38,39,40,43]. Multiple studies have consistently documented the ubiquitous presence of these fungi on stone monuments in the Mediterranean Basin, recognizing them as one of the most destructive microbial groups responsible for the deterioration and irreversible damage inflicted upon these assets [44,45,46,47,48,49,50,51,52,53,54,55,56,57,58]. When colonizing rocks, these fungi can induce chemical deterioration by secreting siderophore-like compounds [59]. However, the most significant damage is believed to result from their hyphal mechanical action, leading to exfoliation and biopitting [60,61]. Furthermore, they cause aesthetical damage as a consequence of melanin production [50,56]. The slow growth and weak competitive ability of these peculiar BF outside their extreme environment turn these unfavorable stone substrates into an ideal habitat for their colonization [58], potentially acting as a reservoir of undiscovered taxa with unknown implications for biodeterioration.

The Mediterranean region is renowned for its extensive cultural heritage, presenting a challenging task of preservation and conservation for the countries within this area [58]. Portugal, with its diverse and historically rich background, is not an exception, featuring numerous exceptional stone-built artworks and monuments throughout its territory. Knowledge of deteriorating agents is of utmost importance for developing effective conservation strategies and well-executed restoration initiatives, ensuring the long-term preservation of these invaluable cultural assets [20,23]. As part of the ongoing efforts to study the fungal diversity thriving in a deteriorated limestone funerary art piece at the Lemos Pantheon [62,63], a national monument located in Águeda, Portugal, two isolates of an unknown slow-growing microcolonial black fungus were obtained. Thus, the aim of this work was to determine the taxonomic relationships of these strains with close relatives through a multi-locus phylogenetic analysis (Internal Transcribed Spacer (ITS), 28S Large Subunit of ribosomal DNA (LSU), and the RNA Polymerase II Second Largest Subunit (*rpb2*)), coupled with morphological, physiological, and ecological examinations. This integrative analysis revealed that these strains were included within the *Aeminiaceae* family, in the *Mycosphaerellales* order. However, they did not cluster with the only known genus representative in this family. The *Aeminiaceae* family was recently established by Trovão et al. [57] to accommodate a novel monophyletic lineage, distinctly placed from other families in the *Capnodiales* but related to the families *Extremaceae* and *Neodevriesiaceae* (now part of *Mycosphaerellales*, *Capnodiales s. lat*. [33]). To date, this family has been represented by a single known genus and species, *Aeminium ludgeri*. However, based on the findings from our comprehensive analysis, we introduce and describe a new genus, *Saxispiralis* gen. nov., and a new species, *Saxispiralis lemnorum* sp. nov., within the *Aeminiaceae* family. Moreover, considering the isolation source of the strains and in an attempt to understand their contribution to the overall stone monument biodeterioration, their *in vitro* biodeteriorative potential was also evaluated. This assessment allowed us to verify their deteriorative ability, shedding light on the potential issues that the proliferation of this fungus could pose under specific conditions for the preservation of this monument.

## 2. Materials and Methods

### 2.1. Site Description, Sample Collection, and Fungal Isolation

The Church of São Salvador da Trofa is a Catholic temple located in Trofa do Vouga (Águeda county), in the district of Aveiro, Portugal. This historic Church houses the Lemos Pantheon, a 16th century burial place built in honor of the Lemos family, one of the most influential families in the region. Designated as a National Monument in 1992, it is an important local landmark and a shining example of Portuguese funerary art [64]. The pantheon comprises two groups of tombstones facing each other, which are carved from white Ançã limestone, a unique type of Portuguese limestone with a relatively high proportion of CaCO_3_ (>96.5%), known for its easy workability and intricate carvings [65,66]. Despite its beauty, the tomb complex shows clear visual signs of biological colonization and biodeterioration in certain areas, causing structural and aesthetic damage to the limestone. Four samples (L1, L2, L3, and L4) were collected indoors in July 2021. Temperature (T) and relative humidity (RH) were monitored at the beginning and end of the sampling procedure using a digital thermohygrometer, with median values of T 22 °C and RH 51%. Samples were collected from areas displaying clear signs of alteration and degradation, using both micro-invasive (scalpel scraping, 0.5 g/site) and non-invasive (nitrocellulose disc swabbing, Ø 5 cm, 2/site) sampling methods, from different types of biodeterioration observed in the tombs. Further details on the sampled areas, including pictures, and sampling procedures can be found in Paiva et al. [62]. Out of a total of 16 isolates obtained from the L4 sample, characterized by abundant salt damage (salt efflorescence), 2 were particularly distinctive and unique. Both isolates were obtained through the suspension of the retrieved sample in 2 mL of sterile 0.9% (*w*/*v*) NaCl solution, vortexed, and plated on Rose Bengal Agar Base (RB, Difco^TM^, Sparks, MD, USA) supplemented with streptomycin (0.5 g L^−1^). Inoculated media plates were incubated in the dark at 25 ± 2 °C for 6 months, and the emerging colonies were transferred to axenic cultures in duplicate, onto Potato Dextrose Agar medium (PDA, Difco^TM^, Sparks, MD, USA) and RB (the medium from which they were originally recovered).

### 2.2. DNA Extraction, PCR Amplification and Sequencing

Genomic DNA was extracted from PDA pure cultures of both isolates using the REDExtract-N-Amp™ Plant PCR Kit (Sigma Aldrich, St. Louis, MO, USA), with several modifications. About 1 mm^3^ of fungal biomass was collected from colonies, placed into 0.2 mL PCR microtubes with 20 μL of extraction solution, and incubated in a thermocycler using the following protocol: 94 °C for 10 min, followed by 60 °C for 13 min and 10 °C for 15 min. After incubation, 20 μL of dilution solution was added, and the resulting mixture was vortexed for 2 min. The obtained DNA was used for PCR amplification, using the primer pairs ITS1-F/ITS4 [67,68], LSU1fd/LR5 [69,70], and frpb2-5F/frpb2-414R [71,72] to amplify three nuclear regions, the Internal Transcribed Spacer (ITS), the 28S Large Subunit of ribosomal DNA (LSU), and a protein coding region, the RNA Polymerase II Second Largest Subunit (*rpb2*). Amplification reactions were performed in 50 μL final volumes and consisted of 25 μL of NZYTaq Green Master Mix (NZYTech™, Lisbon, Portugal), 2 μL of each primer (10 mM), 19 μL of ultra-pure water, and 2 μL of template DNA, using an ABI GeneAmp™ 9700 PCR System (Applied Biosystems, Waltham, MA, USA), with the following conditions: initial denaturation temperature of 96 °C for 2 min, followed by 40 cycles of denaturation temperature of 96 °C for 45 s, primer annealing at 54 °C (ITS), 52 °C (LSU), 49 °C (*rpb2*), primer extension at 72 °C for 90 s, and a final extension step at 72 °C for 2 min. The obtained amplicons were purified with the EXO/SAP Go PCR Purification Kit (GRISP, Porto, Portugal) according to the manufacturer’s protocol and sent for bidirectional Sanger sequencing, using an ABI 3730xl DNA Analyzer system (96 capillary instruments), at STABVIDA, Portugal.

### 2.3. Phylogenetic Analysis

Sequence reads were quality checked using Chromas v.2.6.6 (Technelysium, Southport, QLD, Australia) and aligned and assembled using BioEdit Sequence Alignment Editor^©^ v.7.2.5 (https://bioedit.software.informer.com/download/ (accessed on 20 April 2023)). The obtained consensus sequences were deposited in the GenBank database with the accession numbers OR081767-OR081768 for ITS, OR081765-OR081766 for LSU, and OR074926-OR074927 *rpb2*. Similarity queries were performed using the obtained sequences against the National Center of Biotechnology Information (NCBI) nucleotide database using a BLASTn search algorithm [73]. Based on the results obtained from this initial assessment of all three regions, it was observed that they exhibited genetic similarity to taxa within the recently described *Aeminiaceae* family. The similarity was approximately 94% for ITS and 88% to 89% for *rpb2*, and the LSU region was the only gene providing a reasonable match of 99% in the BLAST analysis. The second closest matches for all sequences were *Capnobotryella* sp. (88%) for ITS, *Neodevriesia hilliana* (94%) for LSU, and *Cystocoleus ebeneus* (78%) for *rpb2*. Furthermore, environmental sequences obtained from deteriorated granite in the entrance hall of the Palace of Xelmírez in Spain, originating from a dark green biofilm studied by Vázquez-Nion and colleagues (2016) [74], also exhibited a significant ITS Blast result (92% to 93%) and were previously reported as being related to *Aeminiaceae*. To better understand the phylogenetics of the isolated strains, and based on these BLAST results, two datasets were created encompassing all representative sequences within the *Aeminiaceae* family, retrieved from GenBank, along with reference sequences from closely related families and other orders within *Capnodiales*, taking into account all three regions (LSU, ITS, and *rpb2*) (Table 1). For each gene, sequences were individually aligned using the online version of MAFFT v.7 [75] and manually adjusted using UGENE v.1.26.3 [76]. The individual alignments were then concatenated using SeaView v.4 [77]. Prior to the phylogenetic analysis, the model of nucleotide substitution was estimated under the Akaike Information Criterion (AIC) using MrModeltest v.2.3 [78] (dataset 1—LSU nst = 6 rates = propinv, ITS nst = 6 rates = invgamma, and rpb2 nst = 6 rates = gamma; dataset 2—LSU nst = 6 rates = invgamma, ITS nst = 6 rates = invgamma, and rpb2 nst = 6 rates = invgamma). Phylogenetic analysis was conducted considering both Maximum Likelihood (ML) and Bayesian (B) methods. The Maximum Likelihood analysis was conducted using RaxmlGUI v.2.0.0 with 1000 bootstrap replicates [79], while the Bayesian MCMC analysis was performed using MrBayes v.3.2.6 [80], with the following parameters: four runs, ten million generations, heated chain “temperature” of 0.15, trees being saved after every 100 generations, and a stop value of 0.01. Upon the analysis conclusion, Tracer v.1.5 [81] was used to ensure that convergence had been reached. The burn-in phase (25%) was discharged, and the remaining trees were used to calculate the Bayesian posterior probabilities in a 50% majority rule consensus tree that was then viewed and edited in FigTree v.1.2.2 [82]. The trees were rooted with *Cladosporium ramotenellum* (ATCC 36970) for dataset 1 and *Parastagonospora nodorum* (CBS 110109) for dataset 2. All the obtained alignments and phylogenetic trees were deposited in Figshare (https://figshare.com/s/c68a893bc9b8f9f3c5d7, (accessed on 21 June 2023)).

### 2.4. Morphological Characterization

For macro-morphological characterization, strains were cultured on PDA, Malt Extract Agar (Difco^TM^, Sparks, MD, USA) supplemented with 10% NaCl (*w*/*v*) (MEA 10%), Leibniz Institute DSMZ—German Collection of Microorganisms and Cell Cultures 372-Halobacteria medium amended with 10% NaCl (*w*/*v*) (HM 10%), and Dichloran Glycerol Agar (DG 18, Oxoid, Basingstoke, UK) at 25 ± 2 °C for 2 to 6 months [57]. Morphological traits such as colony diameter, mycelium color, texture, and form, as well as other characteristics, were recorded via direct observation of the cultured media plates. For micro-morphological characterization, PDA (Difco^TM^, Sparks, MD, USA) and synthetic low-nutrient agar (SNA), following the recipe by Nirenberg [83], were used and observed directly with a light microscope (Leica DM750 (Leica, Wetzlar, Germany)), as well as using the slide culture technique, and both were photographed with a Leica ICC50W digital camera (Leica, Wetzlar, Germany). At least 50 measurements per structure were considered. Both strains were deposited and preserved in Micoteca da Universidade do Minho (MUM), Braga, Portugal.

### 2.5. Physiological Characterization

Temperature preferences, NaCl tolerance, and pH tolerance were evaluated using protocols adapted from Sterflinger [84] and Selbmann et al. [9]. To determine strain preferences and tolerance ranges for these parameters, culture plates were divided into four sections. Inoculation was performed at the intersection point of both axes, at the center of Petri dishes, using small fragments (approximately 0.5 cm^2^ in size, collected using sterile scalpels) of fungal mycelia from fresh, fully matured pure cultures. Following each analysis, the colony diameter was measured along both axes of the sections (refer to Figure S1). Results from the average diameter measurements and standard errors were calculated and recorded for each temperature, NaCl concentration, and pH value using Microsoft 365^®^ Excel^®^ software version 2208. Data from the pH and NaCl concentration assays were subjected to one-way analysis of variance (ANOVA). Whenever significant differences were found (*p* ≤ 0.05), a post hoc Tukey’s Honestly Significant Difference (HSD) test was used to further elucidate differences among treatments, at a significance level α = 0.05. Data from the temperature assay was subjected to the non-parametric Kruskal–Wallis test. Whenever significant differences were found (*p* ≤ 0.05), Dunn’s post hoc analysis was conducted. All statistical analysis was performed using PAST software (v.4.09 https://past.en.lo4d.com/windows, accessed on 25 August 2023).

Tolerance to UV, high temperature, and cold stress was assessed following the methodology adapted from Rizk et al. [85] and Trovão et al. [57]. Inoculation was conducted in the same manner as previously described for each tested condition. Colonies displaying a diameter >2 mm were considered as positive, indicating their capability to recuperate after exposure to stress.

#### 2.5.1. Temperature Preferences

To determine the optimal temperature growth range, the fungal mycelium was inoculated onto PDA (Difco^TM^, Sparks, MD, USA) plates and incubated at four different temperatures (5 °C, 20 °C, 25 °C, and 29 °C) for two months. The colony diameter was then measured for each temperature, following the procedure outlined above. Each experiment was performed in triplicate.

#### 2.5.2. NaCl Tolerance

To analyze the strain’s salinity preference and tolerance range, the fungal mycelium was inoculated onto DSMZ 372-Halobacteria medium containing increasing concentrations of NaCl, ranging from 0% to 30% in 5% increments. The plates were then incubated at 25 ± 2 °C for two months, after which the colony diameter was measured for each NaCl concentration as described above. Each experiment was performed in triplicate.

#### 2.5.3. pH Tolerance

To access the strain’s ability to grow at different pH values, PDA (Difco^TM^, Sparks, MD, USA) medium with pH values ranging from 5 to 11 in steps of 1 was used. The medium was adjusted to the different pH values using different buffer solutions, following the protocol described by Tiago et al. [86]. The plates were incubated at 25 ± 2 °C for two months, after which the colony diameter was measured for each pH as previously described. The experiment was performed in triplicate.

#### 2.5.4. UV Tolerance

The ability of the strain to survive after exposure to UV-C radiation (253.7 nm) was tested under both wet and dry conditions. The fragments of fungal mycelium were placed in sterile Petri dishes with 1 mL of sterile 0.9% (*w*/*v*) NaCl solution (for wet conditions) and without the saline solution (for dry conditions). The Petri dishes were then positioned at ≈30 cm from the UV source (without lids). After 30 min of exposure, as well as at 1, 2, and 3 h, one treated mycelial piece was removed from each condition (wet and dry) and reinoculated in triplicate onto PDA (Difco^TM^, Sparks, MD, USA) plates. The plates were then incubated at 25 ± 2 °C for one month to record the isolate regrowth ability.

#### 2.5.5. Heat and Cold Stress Tolerance

To examine heat resistance, fragments of fungal mycelium were placed in 2 mL tubes containing 1 mL of sterile 0.9% (*w*/*v*) NaCl solution. These tubes were then placed into a shaking thermoblock and exposed to temperatures ranging from 60 °C to 70 °C in 5 °C increments. For each temperature, 3 tubes were utilized, each containing 3 fragments of ±0.5 cm^2^. After exposure periods of 15, 30, and 60 min at each temperature, one tube was collected, and the treated mycelia were subsequently reinoculated onto PDA (Difco^TM^, Sparks, MD, USA) plates (1 fragment per plate, totaling 3 replicates). The plates were then incubated at 25 ± 2 °C for one month to record the fungus’ regrowth ability. The same experiment was repeated in dry conditions (without NaCl solution).

Cold resistance was assessed in a similar manner. Samples were prepared as described earlier, in both wet and dry conditions, and placed in a freezer at −20 °C. After 1 h, 2 h, and 24 h of cold exposure, the treated mycelia were reinoculated onto PDA (Difco^TM^, Sparks, MD, USA). The plates were kept at 25 ± 2 °C for one month to assess the isolate regrowth ability.

### 2.6. Deteriorative Potential Analysis

As the fungal strains were originally isolated from deteriorated limestone, they were screened for their *in vitro* biodegradative abilities, specifically calcium carbonate (CaCO_3_) dissolution, coupled with the evaluation of media pH alteration (acidification of the culture medium), calcium oxalate crystal formation, and other mineralization development. To assess these abilities, the fungal strains were inoculated in triplicate at the center of Petri dishes (±0.5 cm^2^ agar blocks) on various culture media, as specified in Table 2. The effects were then evaluated after incubating for two months at 25 ± 2 °C. To rule out the possibility of spontaneous events, we conducted parallel incubations of non-inoculated Petri dishes using the same culture media utilized in all biodeterioration plate assays, and the occurrence of such phenomena was not observed. Additionally, for the specific pH variation test in Creatine Sucrose Agar (CREA), *Penicillium brevicompactum* (PL096 from Paica et al. [62]), a known acid producer, was simultaneously inoculated to ensure the medium’s sensitivity to pH alteration and its corresponding color change, serving as a secondary control.

## 3. Results and Discussion

### 3.1. Phylogenetic Analysis

Initial comparisons with the sequences deposited in the NCBI database revealed that the isolates exhibited the highest similarity to representatives of the recently described *Aeminiaceae* family within *Capnodiales*, in all three analyzed regions (ITS, LSU, and *rpb2*). Other BLASTn results showed only distant relationships, with a decrease in similarity ranging from 5% to 10% when comparing the best hits to the second-best result. These findings strongly suggested that the isolates were affiliated with the *Aeminiaceae* family.

**Figure 1:**
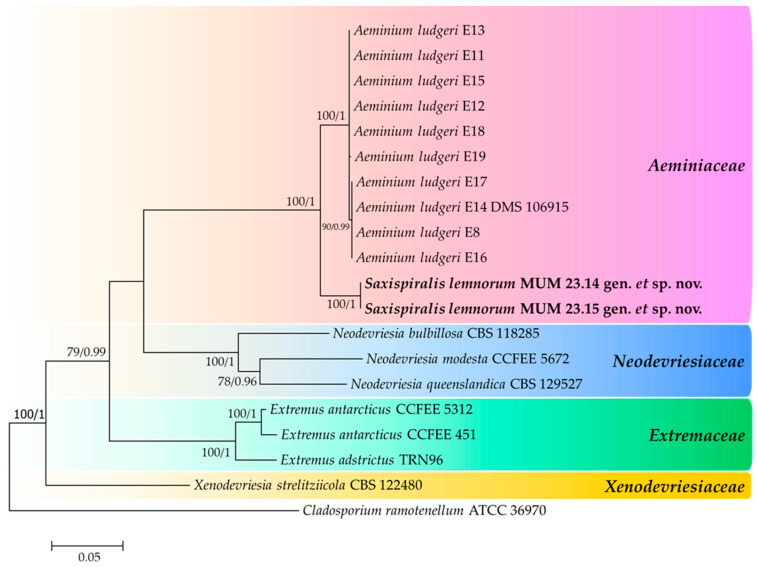
Consensus phylogenetic tree (ML, B) based on a concatenated alignment of LSU/ITS/*rpb2*, including all representative sequences from the *Aeminiaceae* family and selected representatives from closely related families. Families are indicated with colored blocks, and the newly described taxa are highlighted in bold. The scale bar represents the number of substitutions per site, and support values are indicated (>75% bootstrap values for Maximum Likelihood and >0.75 for Bayesian MCMC posterior probabilities). The tree was rooted with *Cladosporium ramotenellum* (ATCC 36970).

The initial phylogenetic analysis was conducted using the aligned sequences of the three concatenated genes, totaling 1388 characters (700 for LSU, 449 for ITS, and 239 for *rpb2*, including alignment gaps). The dataset comprised all 10 representative sequences from the *Aeminiaceae* family, as well as selected representative sequences from the closely related *Extremaceae* and *Neodevriesiaceae* families (Figure 1). The trees generated from Maximum Likelihood and Bayesian analyses exhibited consistent topologies, and both were in accordance with the existing knowledge regarding this family [57]. Furthermore, the phylogenetic analysis demonstrated that the studied strains formed a well-supported monophyletic cluster (100% Bootstrap support and 1.00 Bayesian posterior probability), distinctly placed from *Aeminium ludgeri*, the sole genus and species, previously documented within this family. Based on these findings, we propose the establishment of a new genus, *Saxispiralis* gen. nov., and a new species, *Saxispiralis lemnorum* sp. nov., within the *Aeminiaceae* family to accommodate this fungus.

The traditional monophyletic concept of the *Capnodiales* order was recently redefined by Abdollahzadeh et al. [33] through a multigene phylogeny analysis and lifestyle types. This redefinition involved rearranging the taxa previously classified under *Capnodiales s. lat.*, resulting in the redefinition of *Capnodiales s. str.*, the revival of the *Mycosphaerellales* order, and the introduction of five new orders: *Cladosporiales*, *Comminutisporales*, *Neophaeothecales*, *Phaeothecales*, and *Racodiales*. The proposed *Mycosphaerellales* order comprised eight families, namely *Cystocoleaceae*, *Dissoconiaceae*, *Extremaceae*, *Mycosphaerellaceae*, *Neodevriesiaceae*, *Phaeothecoidiellaceae*, *Schizothyriaceae,* and *Teratosphaeriaceae*. However, the *Aeminiaceae* family was not included in the analysis and therefore it remained associated with the *Capnodiales* order [92]. Nevertheless, since *Extremaceae* and *Neodevriesiaceae* were included in *Mycosphaerellales* and considering their close relationship to *Aeminiaceae*, the latter should also be considered as part of the *Mycosphaerellales* order.

**Figure 2:**
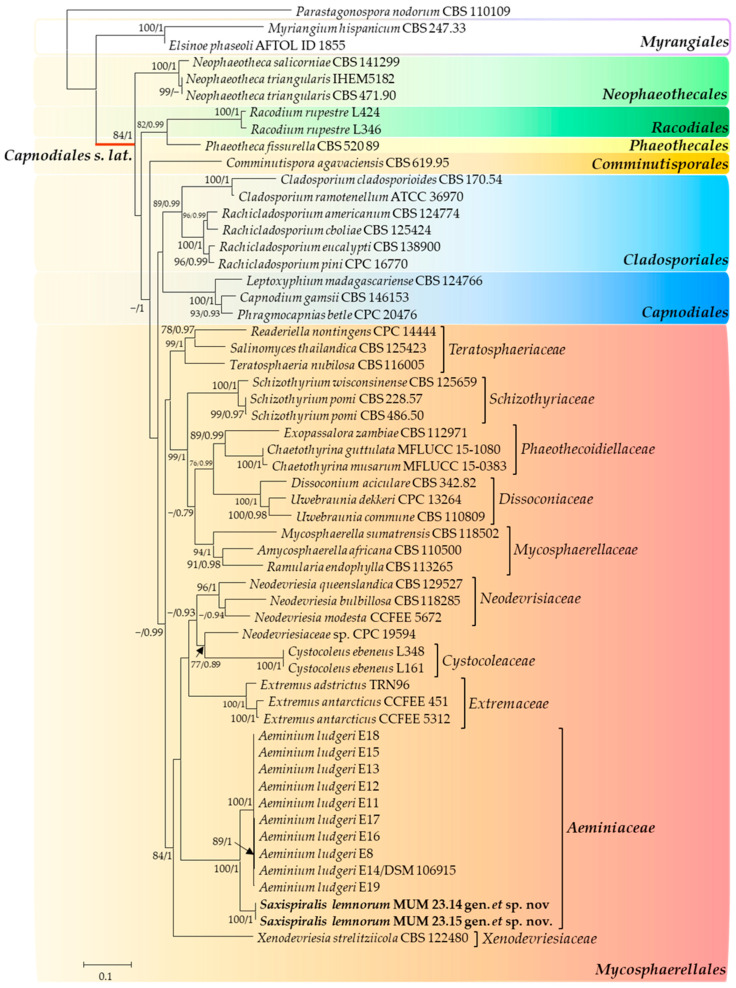
Consensus phylogenetic tree (ML, B) based on a concatenated alignment of LSU/ITS/*rpb2*, containing representative sequences from all families within the *Mycosphaerellales* order, as well as representatives from orders closely related to *Mycosphaerellales*, within *Capnodiales s. lat*. Orders are indicated with colored blocks, and the newly described taxa are highlighted in bold. The scale bar represents the number of substitutions per site, and support values are indicated (>75% bootstrap values for Maximum Likelihood and >0.75 for Bayesian MCMC posterior probabilities). The tree was rooted with *Parastagonospora nodorum* (CBS 110109).

To further clarify the positioning of the *Aeminiaceae* family and the new genus and species proposed in this study, a second analysis was conducted using the aligned sequences of the three concatenated genes, resulting in a total of 1300 characters (681 for LSU, 407 for ITS, and 212 for *rpb2*, including alignment gaps). Following the proposed classification by Abdollahzadeh et al. [33], this second dataset included individuals analyzed in the first dataset, along with representatives of all families associated with the *Mycosphaerellales* order thus far, representatives of orders closely related to *Mycosphaerellales* in *Capnodiales s. lat.*, and representatives of an order outside of *Capnodiales* (*Myrangiales*) (Figure 2). The trees generated from Maximum Likelihood and Bayesian phylogenetic analyses exhibited somewhat similar topologies. Furthermore, the obtained tree aligns with the aforementioned study, providing clear evidence of the placement of the *Aeminiaceae* family within the *Mycosphaerellales* order (100% Bootstrap support and 1.00 Bayesian posterior probability) within *Capnodiales s. lat.* Additionally, the studied strains remained a well-supported monophyletic cluster (100% Bootstrap support and 1.00 Bayesian posterior probability), clearly distinguished from *Aeminium ludgeri* within the *Aeminiaceae* family in the *Mycosphaerellales* order, within *Capnodiales s. lat.* Moreover, Crous et al. [93] introduced *Xenodevriesiaceae* as a new family within *Capnodiales* to accommodate *Xenodevriesia strelitziicola*, a species morphologically similar to *Devriesia* and *Pseudocercospora* (both belonging to the *Teratosphaeriaceae* family) but phylogenetically distinct from both genera. However, the *Xenodevriesiaceae* family was not included in the analysis conducted by Abdollahzadeh et al. [33], and like the *Aeminiaceae* family in the later study conducted by Wijayawardene et al. [92], it also remained associated with *Capnodiales*. Nonetheless, based on the data obtained in this study and in agreement with the available information for this family, our findings suggest not only an affiliation with the *Teratosphaeriaceae* family but also a close relationship with other families within *Mycosphaerellales*, indicating a probable placement of this family within the order. Based on the gathered information, it can be inferred that *Mycosphaerellales* may encompass not just 8, but rather 10 families.

To date, representatives of *Aeminiaceae* family have exclusively been found associated with different deterioration scenarios affecting stone monuments in the Iberian Peninsula, including *Aeminium ludgeri* isolated from limestone at the Old Cathedral of Coimbra [57], environmental samples obtained from granite at the Cathedral of Santiago de Compostela [74], and *Saxispiralis lemnorum* found in Ançã limestone at the Lemos Pantheon of Trofa do Vouga, Águeda. These findings provide important insights into the ecological aspects and potential geographical distribution of this family, suggesting that it may be endemic to the Iberian Peninsula and exclusively inhabit stone substrates.

### 3.2. Taxonomy and Morphological Characterization

**Taxonomy**

*Aeminiaceae* J. Trovão, I. Tiago and A. Portugal

**Figure 3:**
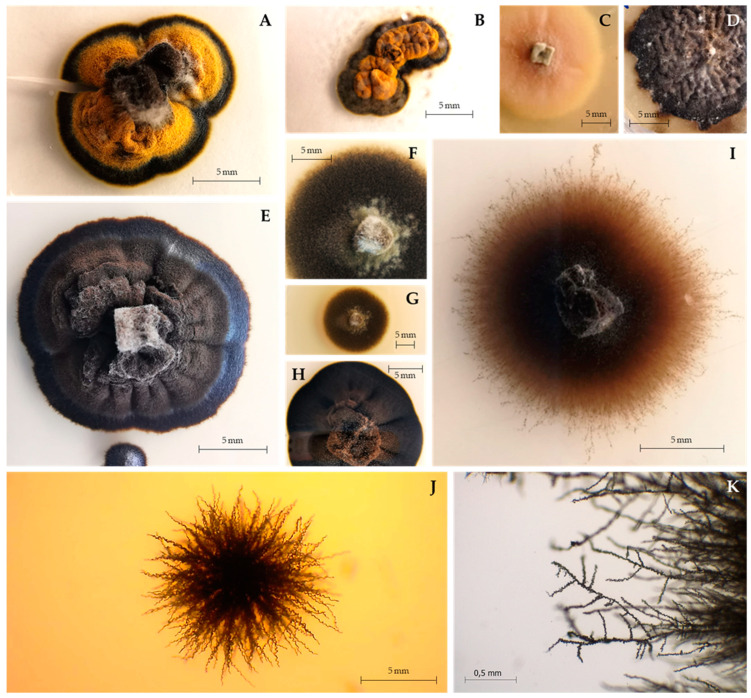
Macromorphology of *Saxispiralis lemnorum*. (**A**,**B**,**E**) Colony appearance on PDA before and after maturation, with melanization progressing to a fully black color. (**C**,**D**) Colony appearance on DG 18 before and after maturation. (**F**,**G**) Colony appearance on HM 10%. (**H**) Colony appearance on MEA 10%. (**I**) Colony appearance on SNA. (**J**,**K**) Detail of the hyphae exhibiting a characteristic spiral-like shape in PDA and SNA, respectively.

**Figure 4:**
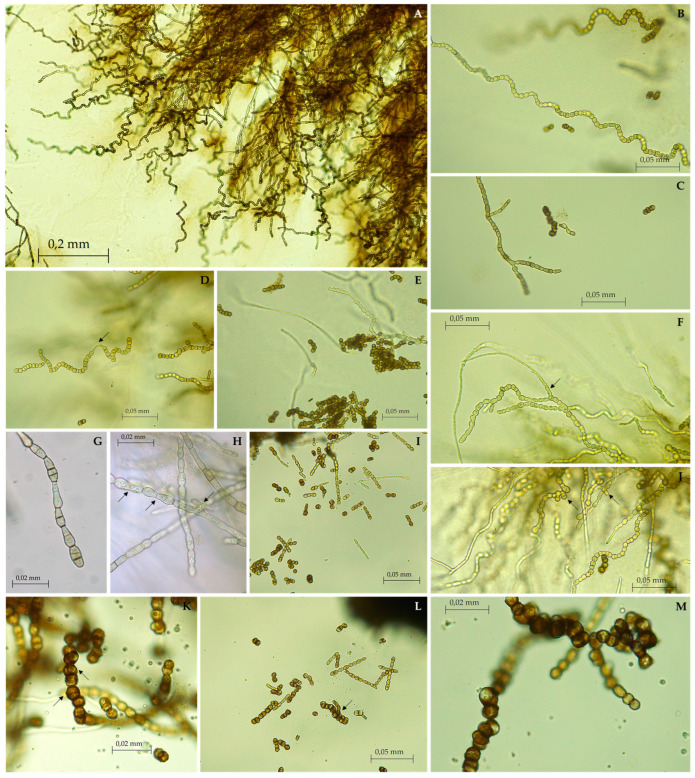
Micromorphology of *Saxispiralis lemnorum*. (**A**,**B**) Unbranched spiral toruloid hyphae with melanized cells. (**C**) Branched mature hyphae. (**D**) Incomplete disarticulation of artic conidia and hyphal fragments remaining joined by connectives (arrow). (**E**) Mature globose conidia. (**F**) Blastic elongation (arrow). (**G**) Filamentous hyphae with intercalary swollen cells terminating in a swollen ellipsoidal cell. (**H**) Dumbbell-shaped hyphal cells with anastomosis (arrows). (**I**) Fragments from different stages of Arthroconidia differentiation. (**J**) Blastic budding (arrows). (**K**) Enlarged meristematic cells subdivided by septations in various directions (arrows). (**L**,**M**) Clumps of mature conidia.

***Saxispiralis*** D.S. Paiva & A. Portugal, **gen. nov.** (Figure 3 and Figure 4).

**MycoBank number:** MB849259.

**Etymology:** «Saxi» (L. neut. n. saxum, genitive) derives from the Latin word for stone, reflecting the substratum from which the strain was isolated; «spiralis» (L. fem. adj.) is derived from the Latin word for spiral, alluding to the distinctive spiral shape of its hyphae.

**Type species:** *Saxispiralis lemnorum* D.S. Paiva & A. Portugal.

**Description:** Monotypic genus to accommodate a novel fungal species in the *Aeminiaceae* family, *Mycosphaerellales* order. Filamentous, slow-growing, anamorphic fungus. Mycelium consisting of cylindroid, pale brown, smooth, septate, and often branched hyphal cells. Prior to fragmentation into arthroconidia, the cells gradually become swollen (torulose), thick-walled, and darker and develop into long meristematic terminal chains of conidia. The conidia are dark brown, thick-walled, rugose, and globose. The arthric disarticulation of hyphae creates a spiral-like shape in the conidial chains, which is unique to this genus and is not reported in any phylogenetically close relatives. Chlamydospores were not observed in culture, and a recognizable sexual morph is absent.

***Saxispiralis lemnorum*** D.S. Paiva & A. Portugal, **sp. nov.** (Figure 3 and Figure 4).

**MycoBank number:** MB849260.

**Etymology:** «Lemnorum» (derived Latinization of Portuguese “Lemos” to Latin “Lemnos”, genitive plural), in honor of the Lemos family, as the strain was found in the Lemos Pantheon.

**Typification:** PORTUGAL, Aveiro, Águeda (40°36.653′ N, 08°28.729′ W), isolated from a deteriorated funerary art piece carved in Ançã limestone with abundant salt damage, in the Lemos Pantheon, 8 June 2021, D.S. Paiva, (holotype MUM-H 23.14, dried specimen), ex-type culture MUM 23.14.

**Description:** Hyphae subhyaline to pale brown when young, consisting of cylindroid cells, guttulate, thin- and smooth-walled, branched, 2.91 ± 0.44 μm wide, occasionally terminating in a swollen ellipsoidal cell, with anastomoses often observed. Prior to maturation, hyphae gradually become swollen, dumbbell-shaped, constricted at the septum, thick-walled, and darker, and develop into long spirals of meristematic torulose conidial chains. Arthroconidia dark brown, thick-walled, verrucose or coarsely punctate, spherical, measuring 5.89 ± 0.52 µm, guttulate, resulting from the differentiation of terminal toruloid-like hyphal cells. Terminal and lateral blastic budding cells are frequently produced, developing longitudinal and oblique septa, and sometimes forming multicellular clumps. Occasionally, conidial secession is incomplete, leaving adjacent cells interconnected by narrow and pale connectives. Chlamydospores absent. Teleomorph unknown.

**Colony characteristics:** On PDA at 25 °C, colonies grew slowly, reaching 13.4 mm in diameter after 30 days and up to 24.8 mm after 2 months. Mycelium with fine velvety texture, dark greyish olive on the reverse (according to the ISCC-NBS Colour System No. 111) and strong orange-yellow color (ISCC-NBS No. 68) with a dark greyish olive margin (No. 111) on the obverse. After 2 months or longer, colonies reached full maturation and became completely melanized and black (ISCC-NBS No. 267) on both sides of the culture. Colonies raised centrally, cerebriform, flat near the periphery and partially immersed in the agar, compact, stiff, lobed, with well-defined regular margin.

Colonies on MEA 10% at 25 °C exhibited a similar morphology to those on PDA, with slight differences. No color alteration was observed; mycelium grew directly brownish black (ISCC-NBS No. 65) both on the reverse and obverse. Colonies were less folded, circular, velvety, with a slightly moist appearance, attaining a diameter of 12 mm in 30 days and up to 35 mm in two months.

On DG-18 agar, at 25 °C, colonies exhibited slow to moderate growth, reaching 21 mm in diameter after 30 days and attaining 32.7 mm after two months, circular, flat, margin entire and slight submerged, velvety, with a slightly moist appearance and covered by sparse whitish aerial mycelium, with a pale orange-yellow color (ISCC-NBS No. 73) on the obverse and reverse. After 2 months or longer, colonies reached full maturation and became completely melanized with a brownish-black color (ISCC-NBS No. 65) on both sides of the culture, becoming raised, rugose, with short aerial hyphae present, and undulate margin.

Colonies on HM 10% at 25 °C showed slow to moderate growth, reaching 26 mm in diameter after 30 days and up to 41 mm after two months, dark greyish olive in the reverse (ISCC-NBS No. 111) and dark olive (ISCC-NBS No. 108) on the obverse, becoming white (ISCC-NBS No. 263) near the periphery, circular, flat, with entire margin, producing thin whitish aerial mycelium.

**Substrate:** Ançã limestone.

**Distribution:** Portugal.

**Additional specimens examined:** PORTUGAL, Aveiro, Águeda (40°36.653′ N, 008°28.729′ W), isolated from a deteriorated funerary art piece carved in Ançã limestone with abundant salt damage, in the Lemos Pantheon, 8 June 2021, D.S. Paiva, MUM 23.15.

**Figure A1:**
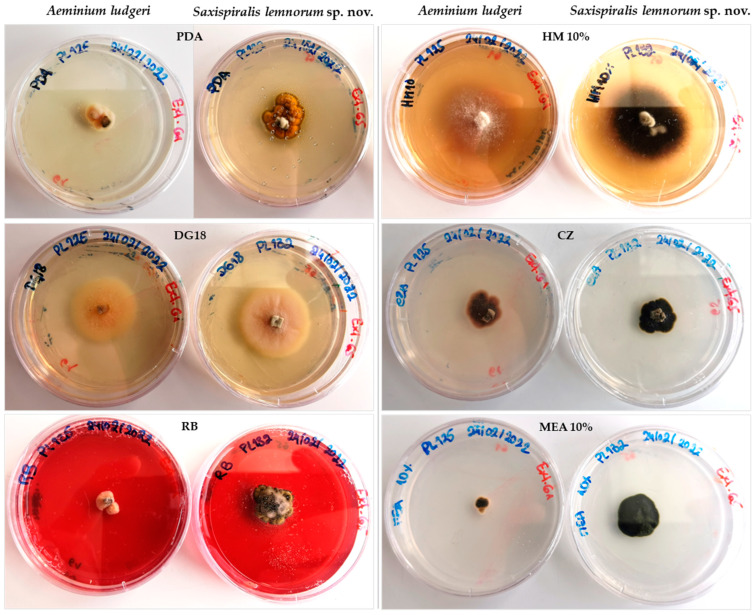
Direct comparison of the macromorphology between *Aeminium ludgeri* (PL125 isolate, also obtained during the same study and from the same sample [61]) and *Saxispiralis lemnorum* is shown on different culture media after one month of inoculation (Ø 5 cm plates). PDA—Potato Dextrose Agar (PDA); DG 18—Dichloran-Glycerol Agar; RB—Rose Bengal Agar; HM 10%—DSMZ 372-Halobacteria medium supplemented with 10% NaCl (*w*/*v*); CZ*—Czapek Solution Agar; MEA 10%—Malt Extract Agar supplemented with 10% NaCl (*w*/*v*). * Culture medium used in Paiva et al. [61] for processing the initial samples.

**Figure A2:**
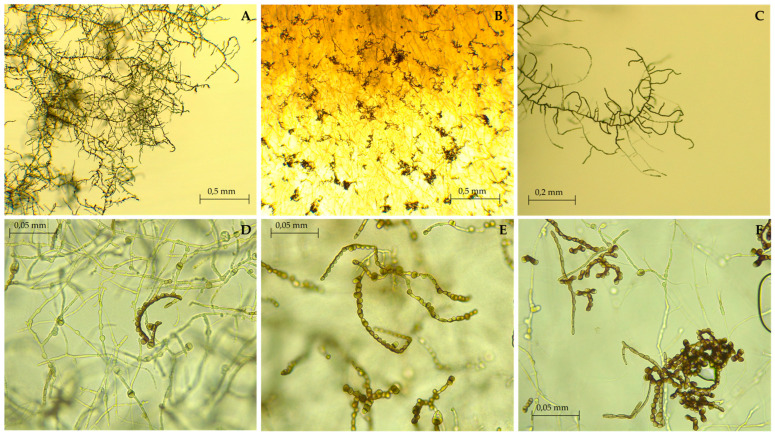
Micromorphology of *Aeminium ludgeri* (PL125 isolate) shown as a reference for comparison with *Saxispiralis lemnorum*. (**A**–**C**) Differentiated, toruloid-like hyphae and mature chains of arthroconidia. (**D**–**F**) Micronematous intercalary and terminal conidial chains.

**Notes:** The newly identified species *Saxispiralis lemnorum* exhibits distinct phylogenetic and morphological characteristics that set it apart from other previously described members of the *Aeminiaceae* family. Phylogenetic analysis based on the concatenated ITS rDNA, LSU, and *rpb2* dataset considered in the present study revealed that the retrieved strains form a distinct monophyletic lineage within the *Aeminiaceae* family, clearly separate from the previously known genus *Aeminium*, which, until now, represented the sole genus in this family. Phenotypically, it can be easily distinguished from *A. ludgeri* by its distinct colony color and hyphal characteristics in terms of shape and structure (Appendix A, Figure A1 and Figure A2), as well as its larger size with pronounced rough texture conidia, which are exclusive to this genus.

### 3.3. Physiological Characterization

**Figure 5:**
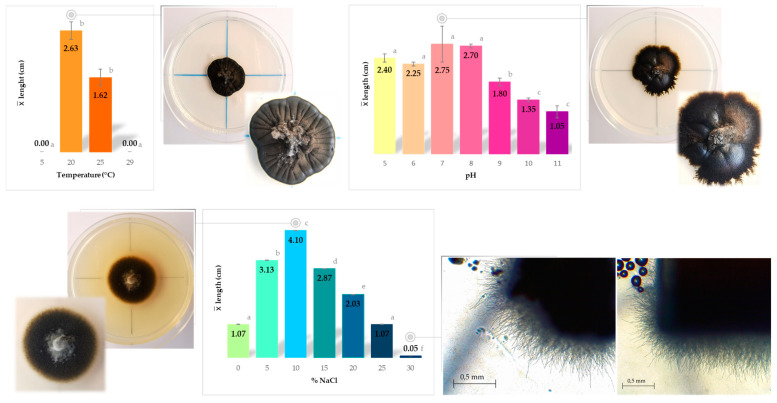
Temperature, pH, and salinity preferences of *Saxispiralis lemnorum* represented by the colony diameter reached after two-month incubation at 25 ± 2 °C. Temperature and pH tests were conducted in PDA medium, while salinity preference was evaluated using DSMZ 372-Halobacteria medium. Results linked by the same letter do not exhibit significant differences (α = 0.05).

Physiological analyses were conducted on both isolates obtained in this study. However, no significant differences were observed among the isolates across the various tested conditions. The summary of the results from physiological characterization for temperature, pH, and salinity preference/tolerance is presented in Figure 5. The data obtained serve as a comprehensive overview of the strain’s responses to temperature, pH, and salinity, enabling a better understanding of their ecological preferences and adaptability.

Based on the observed growth patterns at the tested conditions, the strains exhibited optimal growth at moderate temperatures, particularly at 20 °C, where the colony diameter reached 2.63 cm after 2 months. At 25 °C, a slight decrease in growth was observed, and no growth was recorded at lower and higher temperatures. Considering these findings, can be inferred that the fungus displays a preference for mesophilic conditions.

Regarding the pH assessment, the strains displayed a wide range of tolerance, as they grew across a broad spectrum of pH levels, ranging from acidic (pH 5.0) to alkaline (pH 11.0). Optimal growth was observed between pH 7.0 and pH 8.0, while the lowest growth was recorded at pH 11.0. Although the optimum pH for growth was pH 7, there was only a slight decrease in growth at the more acidic pH values of 5 and 6, whereas a more significant decrease was observed at higher alkaline pH values (pH 11). These results indicate that the fungus can be classified as highly tolerant to pH fluctuations, showcasing the ability to thrive across a wide pH spectrum without experiencing severe growth impairments. This distinct characteristic sets it apart from *Aeminium ludgeri*, the other member in *Aeminiaceae* family, considered a facultative alkaliphile, with no growth being registered for pH levels below 6 and above 9 [57].

The isolates exhibited growth across the entire range of tested NaCl concentrations, from 0% up to 30%. Optimal growth was observed at a concentration of 10% NaCl. Growth was still observed at 30% NaCl, albeit at a slower rate (>2 mm diameter after 3-month post-inoculation). Among halotolerant fungi, *Hortaea werneckii* is currently considered the most halotolerant fungus known to date, as it can tolerate a wide range of NaCl concentrations from 0% to 32%, with an optimal growth range of 6% to 14% NaCl [17,94]. Additionally, a recently discovered new ecotype of *Pseudotaeniolina globosa* [85] has also demonstrated the ability to grow at up to 30% NaCl, placing *Saxispiralis lemnorum* alongside these notable “kings” of salinity. The fungus’ capacity to develop in diverse NaCl concentrations assumes particular significance when considering its original isolation from a sample collected in an environment characterized by pronounced salt damage. This environmental context aligns with its ability to thrive under high-salinity conditions. Based on this characteristic, the fungus can be classified as halotolerant.

After subjecting the fungus to thermal stress, no growth was observed for any of the tested conditions following the heat tolerance protocol. Consequently, the fungus was classified as non-heat tolerant. On the other hand, when exposed to low-temperature shock, across all tested conditions, the fungus consistently demonstrated recovery and growth, indicating its cold-tolerant nature or psychrotrophic adaptation. A similar pattern was observed when subjected to UV radiation stress, as it exhibited the ability to recover and grow under all tested conditions. Additionally, based on its successful growth on DG-18 culture medium, which was used for morphological characterization, the fungus can be considered as xerophilic.

The remarkable ability of this new species to tolerate a wide variety of physicochemical parameters and withstand different types of stress suggests an extremotolerant nature. Furthermore, the gathered data provide strong evidence that *Saxispiralis lemnorum* is well-suited for survival on stone surfaces, where high evaporation rates and low water availability lead to local increases in ion concentrations and fluctuations in pH, creating an environment to which it can successfully adapt.

### 3.4. Deteriorative Potential

When evaluating heritage items, it is imperative to not only assess the diversity and viability of microorganisms but also conduct complementary studies on physiological activities and their deteriorative potential. These additional studies are crucial for gaining a comprehensive understanding of the occurring biodeterioration processes and developing tailored and effective treatment strategies [88]. As the isolates were derived from deteriorated limestone, the evaluation of their deteriorative potential was determined by assessing their ability to induce pH alteration, CaCO_3_ dissolution, and mineral production. The overall results for the biodegradative plate assays considered in this study can be found in Table 3.

**Figure A3:**
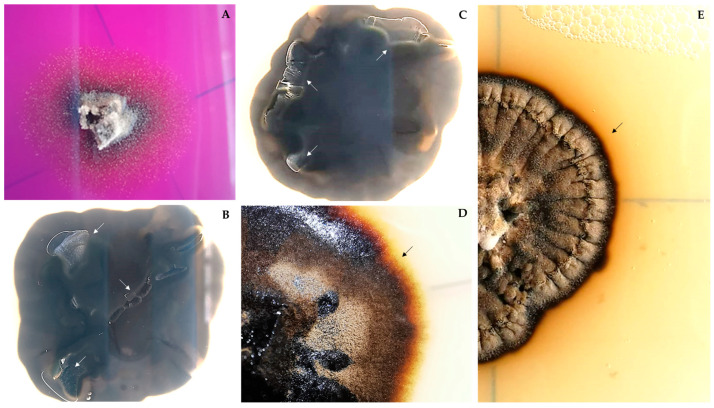
Observable alterations caused by *Saxispiralis lemnorum* in different culture media. (**A**) Poor growth and absence of acid reactions on CREA medium. (**B**,**C**) Hyphal intrusion resulting in the fragmentation of the PDA medium. (**D**,**E**) Noticeable production of dark yellow pigment in MEA media adjusted to pH 10 and B4.

The presence of fungi on the stone surface can have a profound impact on the material properties due to the secretion of inorganic and organic acids as a byproduct of their metabolic processes [18,19,20,23,25,26]. Carbonate dissolution primarily occurs through the action of organic acids [18,20,23]. However, our results demonstrate that the fungal strains did not induce detectable pH alterations on CREA medium (Appendix A, Figure A3A) and exhibited no ability to dissolve CaCO_3_. In most cases, the production and release of acids into the substrate are particularly favored when fungi inhabit nutrient-rich substrates that promote rapid growth [95]. This intensive growth leads to the excessive production of organic acids beyond the necessary requirements for normal metabolism, which are subsequently excreted into the substrate, rendering them detectable [96]. Therefore, our findings are consistent with expectations, considering that *Saxispiralis lemnorum* is a microcolonial fungus characterized by slow growth and adaptation to harsh oligotrophic substrates. Furthermore, this is in line with current knowledge regarding MCF, as chemical deterioration of stone by acid substances has never been demonstrated for this group of fungi [20,26,97].

**Figure 6:**
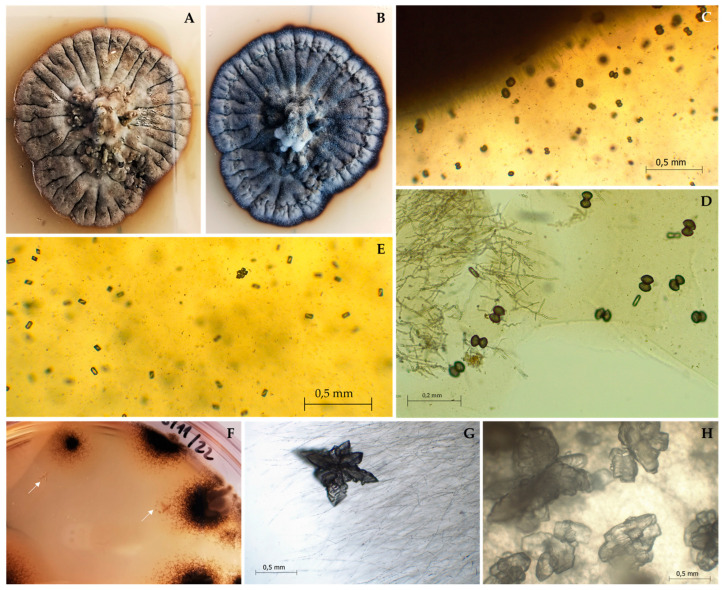
Mycogenic minerals formed on inoculated B4 and HM10% media. (**A**,**B**) Colony appearance on B4 medium, before and after maturation (black mycelium), respectively. (**C**–**E**) Different shapes of precipitated crystals on B4 medium: spherical clusters and combined forms of tetragonal prisms. (**F**) Direct visualization of the HM 10% culture plate displaying precipitated crystals. (**G**,**H**) Platy crystal aggregates.

Fungal biological activities can release cations from minerals and lead to the formation of secondary minerals through a process called biomineralization [21,24,25,26,98]. This adaptation is particularly observed in fungi growing on calcium-rich substrates [99], and such mineralization events can contribute to various biodeterioration phenomena [18,100]. Biogenic crystal formation was observed in the B4 and HM 10% media with different configurations (Figure 6). In the B4 medium, spherical crystal formations were abundant around the fungal mycelium and surrounding areas (Figure 6C,D), while tetragonal forms were predominantly found at the edge of the Petri dish (Figure 6E). Irregularly shaped platy crystal aggregates were observed in the HM 10% medium, typically found beneath or near the fungal mycelium (Figure 6F–H). A possible explanation for the observed crystallization phenomenon in the B4 medium, but not in media containing calcium carbonate, could be attributed to fungal respiration activity, supplying the required CO_2_ for calcite precipitation. In the B4 medium, the release of calcium does not necessarily rely on the fungi producing organic acids, since calcium acetate is highly soluble, and calcium is readily available in the solution. However, in the MB4 medium (e.g.), which contains nearly insoluble calcium carbonate, the presence of organic acids becomes necessary to facilitate the release of calcium [96].

However, since crystals of different conformations exist in the same medium, further studies would be necessary to characterize their composition and understand the actual mechanisms involved in their formation. The same applies to the crystals present in HM 10%. However, for this latter case, it is important to note that although this medium was used with various salt concentrations in the NaCl tolerance assay, crystallization was only observed at a 10% NaCl concentration, which is the optimal growth concentration. Crystallization was never observed at lower or higher concentrations. Based on the obtained results, *Saxispiralis lemnorum* has demonstrated the ability for secondary mineral formation, suggesting its potential involvement in the promotion of significant stone biodeterioration. The formation of crystals on the stone matrix can result in pore and fissure expansion, efflorescence formation, as well as peeling and spalling of the materials [101], all of which are consistent with the characteristics observed in the sampled area. Therefore, the proliferation of this species, influenced by specific environmental factors, is likely to contribute to the observed deterioration phenomena in L4.

Another interesting observation made with some of the media used for characterizing the isolates, e.g., PDA, was the presence of fungal growth not only on the surface, but also hyphae growing within and through the medium. In many cases, this led to the medium cracking and detaching from the Petri dish (Appendix A, Figure A3B,C). This could be an indication of its potential to cause physical biodeterioration resulting from the mechanical forces generated by hyphal penetration, which led to the breakage of the medium. MCF have been consistently reported as capable of physically penetrating stone substrates through the combined action of mechanical forces, exerted by hyphal expansion during growth, and chemical processes involving metal chelating compounds [26,95,97,101]. This penetration process can lead to the loosening of the intercrystalline stone matrix due to internal pressure, ultimately resulting in detachment and material loss [96,101]. Taking this into consideration, along with the aforementioned ability for secondary mineral formation, this species is likely to actively contribute to the observed alterations in the sampled area.

Furthermore, in the B4 medium, as well as in the more alkaline media used in the pH tolerance assay (pH 9 to 11), the presence of a dark yellow pigment was observed (Appendix A, Figure A3D,E). Along with its highly melanized mycelium, which gives it a black color, these are indicators that these strains may cause chromatic alterations due to mycelial growth and pigment production.

## 4. Conclusions

Our study reports a newly discovered microcolonial black fungus belonging to the *Aeminiaceae* family, which had only one known representative until now. The fungal isolates here described represent a novel genus and species, found as part of a complex community colonizing deteriorated limestone in the Lemos Pantheon, highlighting the need for further investigation of fungal communities in similar environments. This work provides valuable molecular, morphological, and physiological data that enhance our understanding of fungi in this recently described family. Furthermore, the physiological and biodeteriorative potential assessment of this new species yielded data that demonstrate a significant deteriorative activity, indicating its potential to harm the substrate and cause various types of alterations. Further studies are currently underway to fully understand its nature and abilities. Understanding the microbial agents involved in the materials’ biodeterioration is crucial knowledge for appropriate safeguarding measures to be considered, discussed, and implemented for effective conservation. By comprehensively studying the microbial agents responsible for material deterioration, we can hope to preserve our cultural heritage for future generations.

## Figures and Tables

**Table 1:** List of reference isolates considered in the phylogenetic analyses and their respective GenBank accession numbers. The newly generated sequences are highlighted in bold for reference.

Species	Strain ID ^1,2^	Country	Substrate	GenBank Accession Number ^3^		
				LSU	ITS	*rpb2*
*Aeminium ludgeri*	E8	Portugal	Limestone	MG938284	MG938056	MG948613
*Aeminium ludgeri*	E11	Portugal	Limestone	MG938285	MG938057	MG948614
*Aeminium ludgeri*	E12	Portugal	Limestone	MG938286	MG938054	MG948615
*Aeminium ludgeri*	E13	Portugal	Limestone	MG938287	MG938061	MG948616
*Aeminium ludgeri*	DSMZ 106916 ^T^	Portugal	Limestone	MG938288	MG938062	MG948617
*Aeminium ludgeri*	E15	Portugal	Limestone	MG938289	MG938063	MG948618
*Aeminium ludgeri*	E16	Portugal	Limestone	MG938290	MG938055	MG948619
*Aeminium ludgeri*	E17	Portugal	Limestone	MG938291	MG938058	MG948620
*Aeminium ludgeri*	E18	Portugal	Limestone	MG938292	MG938059	MG948621
*Aeminium ludgeri*	E19	Portugal	Limestone	MG938293	MG938060	MG948622
*Amycosphaerella africana*	CBS 110500 ^ET^	Australia	*Eucalyptus globulus*	KF901837	KF901516	KF902223
*Capnodium gamsii*	CBS 146153	Thailand	*Lagerstroemia speciosa*	MN749168	MN749238	MN829263
*Chaetothyrina guttulata*	MFLUCC15-1080	Thailand	–	KU358917	KX372277	–
*Chaetothyrina musarum*	MFLUCC15-0383	Thailand	–	KU710171	KX372275	–
*Cladosporium cladosporioides*	CBS 170.54 ^NT^	UK, England	*Arundo* leaf	MH868815	AY213640	GU357790
*Cladosporium ramotenellum*	ATCC 36970	–	*Populus tremuloides*	MF951116	MF951281	MF951413
*Comminutispora agavaciensis*	CBS 619.95 ^T^	USA	Plant	EU981286	MH862543	MN829337
*Cystocoleus ebeneus*	L161	Austria	Rock sample	EU048578	–	–
*Cystocoleus ebeneus*	L348	Austria	Rock sample	EU048580	–	–
*Dissoconium aciculare*	CBS 342.82	Germany	–	EU019266	AF173308	–
*Elsinoe phaseoli*	CBS 165.31	Cuba	*Paseolus lunatus*	DQ678095	KX887263	KX887144
*Exopassalora zambiae*	CBS 112971 ^T^	Zambia	*Eucalyptus globulus*	EU019273	AY725523	MF951421
*Extremus adstrictus*	TRN 96 ^ET^	Spain	Rock	KF310022	AY559346	KF310103
*Extremus antarcticus*	CCFEE 5312	Antarctica	Rock	KF310020	KF309979	KF310086
*Extremus antarcticus*	CCFEE 451	Antarctica	Sandstone	GU250360	KF309978	KF310085
*Leptoxyphium madagascariense*	CBS 124766 ^T^	Madagascar	*Eucalyptus* sp.	MH874923	GQ303277	MN829296
*Mycosphaerella sumatrensis*	CBS 118502	Indonesia	*Eucalyptus* sp.	KF901996	JX901775	KF902222
*Myriangium hispanicum*	CBS 247.33	–	*Acer monspessulanum*	KX887067	MH855426	GU371744
*Neodevriesia bulbillosa*	CBS 118285	Spain	Rock	KF310029	AY559341	KF310102
*Neodevriesia modesta*	CCFEE 5672 ^ET^	Italy	Rock	KF310026	NR_144975	KF310093
*Neodevriesiaceae* sp.	CPC 19594	Brazil	Mycoparasite	KJ564327	–	KJ564349
*Neodevriesia queenslandica*	CBS 129527	Australia	*Scaevola taccada*	KF901839	JF951148	KF902234
*Neophaeotheca salicorniae*	CBS 141299	Africa	*Salicornia meyeriana*	MH878214	KX228276	MN829343
*Neophaeotheca triangularis*	CBS 471.90 ^T^	Belgium	Humidifier	NG057776	–	MN829344
*Neophaeotheca triangularis*	IHEM 5182	Belgium	Humidifier	MH873909	–	–
*Parastagonospora nodorum*	CBS 110109	Denmark	*Lolium perenne*	EU754175	KF251177	KF252185
*Phaeotheca fissurella*	CBS 520.89 ^T^	Canada	*Pinus contorta*	MH873872	MH862184	MN829342
*Phragmocapnias betle*	CPC 20476	Philippines	Palm	MN749222	MN749294	MN829324
*Rachicladosporium americanum*	CBS 124774 ^T^	USA	Leaf litter	GQ303323	GQ303292	MN829336
*Rachicladosporium cboliae*	CBS 125424 ^T^	USA	Twig debris	MH875168	GU214650	LT799763
*Rachicladosporium eucalypti*	CBS 138900 ^T^	Ethiopia	*Eucalyptus globulus*	NG070537	NR155718	–
*Rachicladosporium pini*	CPC 16770	Netherlands	*Pinus monophylla*	JF951165	JF951145	LT799764
*Racodium rupestre*	L346	Austria	Rock sample	EU048583	GU067666	–
*Racodium rupestre*	L424	Italy	Rock sample	EU048582	GU067669	–
*Ramularia endophylla*	CBS 113265 ^ET^	Netherlands	*Quercus robur*	KF902072	KF901725	KF902358
*Readeriella nontingens*	CPC 14444	Australia	*Eucalyptus oblonga*	KF902073	KF901726	KF902378
*Salinomyces thailandica*	CBS 125423	Thailand	*Syzygium siamense*	KF902125	GU214637	KF902206
***Saxispiralis lemnorum* sp. nov.**	**MUM 23.14**	**Portugal**	**Limestone**	**OR081765**	**OR081767**	**OR074926**
***Saxispiralis lemnorum* sp. nov.**	**MUM 23.15**	**Portugal**	**Limestone**	**OR081766**	**OR081768**	**OR074927**
*Schizothyrium pomi*	CBS 486.50	Netherlands	*Polygonum sachalinense*	KF902024	–	KF902385
*Schizothyrium pomi*	CBS 228.57	Italy	–	KF902007	–	KF902384
*Schizothyrium wisconsinense*	OH49A1c	USA	Apple fruit	FJ147158	FJ425209	–
*Teratosphaeria nubilosa*	CBS 116005	Australia	*Eucalyptus globulus*	KF902031	KF901686	KF902460
*Uwebraunia commune*	CPC 830 ^ET^	South Africa	*Eucalyptus nitens*	KJ564336	–	KJ564351
*Uwebraunia dekkeri*	CPC 13264	Australia	*Eucalyptus molucana*	GQ852593	–	KJ564340
*Xenodevriesia strelitziicola*	CBS 122480	South Africa	*Strelitzia* sp.	GU214417.1	GU214635.1	–

^1^ ATCC: American Type Culture Collection, Virginia, USA; BCCM/IHEM: Belgian Coordinated Collections of Microorganisms/Fungi Collection: Human & Animal Health, Sciensano, Brussels, Belgium; CBS: Westerdijk Fungal Biodiversity Institute, Utrecht, The Netherlands; CCFEE: Culture Collection of Fungi from Extreme Environments, Dept. of Ecological and Biological Sciences, University of Tuscia, Viterbo, Italy; CPC: Collection Pedro Crous, housed at CBS; DSMZ: German Collection of Microorganisms and Cell Cultures GmbH, Leibniz Institute, Germany; MFLUCC: Culture collection of Mae Fah Luang University (MFLU), Chiang Rai, Thailand; MUM: Micoteca da Universidade do Minho, Centre of Biological Engineering, University of Minho, Portugal; TRN: T. Ruibal personal collection. ^2^ ET: ex-type; NT: ex-neotype strain; T: type. ^3^ ITS: Internal transcribed spacer region; LSU: Large subunit of the 28S nrRNA; *rpb2*: partial DNA-directed RNA polymerase II second largest subunit gene.

**Table 2:** Biodeteriorative plate assays.

Analyses	Culture Medium	Composition *	Final pH	Positive Reaction	Reference
CaCO_3_ dissolution	CaCO_3_ Glucose Agar (CGA)	5 g CaCO_3_, 5 g glucose, and 15 g agar in 1 L deionized water	7	Visualization of a clear zone around the colony	[87,88]
Calcium oxalate crystal formation	Modified Malt Extract Agar (MMA)	33.6 g MEA and 5 g/L of CaCO_3_ in 1 L deionized water	7	Observation of calcium oxalate crystals under the light microscope	[88,89]
Other mineral precipitation	B4	10 g calcium acetate, 5 g yeast extract, 5 g glucose, and 15 g agar in 1 L deionized water	8	Observation of mineral precipitation (crystals) under the light microscope	[63,88,90]
	Modified B4 (MB4)	Same recipe as B4 medium with substitution of calcium acetate for CaCO_3_	8		
	HM 10%	5 g yeast extract, 5 g casamino acids, 1 g Na-glutamate, 2 g KCl, 3 g Na_3_-citrate, 20 g MgSO_4_·7H_2_O, 100 g NaCl, 36 g FeCl_2_·4H_2_O, 0.36 mg MnCl_2_·4H_2_O, and 20 g agar in 1 L deionized water	8		
pH alteration	Creatine Sucrose Agar (CREA)	3 g creatine, 30 g sucrose, 1.3 g K_2_HPO_4_·3H_2_O, 0.5 g MgSO_4_·7H_2_O, 0.5 g KCl, 0.01 g FeSO_4_·7H_2_O, 0.01 g ZnSO_4_·7H_2_O, 0.005 g CuSO_4_·5H_2_O, 0.05 g Bromocresol purple, and 15 g agar in 1 L deionized water	8	Visualization of a yellow halo around the colony	[88,91]

* All culture media components were sourced from VWR (Radnor, PA, USA), except for Malt Extract Agar (MEA), which was from Difco^TM^ (Sparks, MD, USA), and Bromocresol purple, which was from Sigma Aldrich (St. Louis, MO, USA).

**Table 3:** Biodeterioration assays.

*Saxispiralis lemnorum* gen. *et* sp. nov. Isolates	Acid Production	CaCO_3_ Dissolution	Calcium Oxalate Crystal Formation	Other Mineral Precipitation		
	CREA ^1^	CGA ^1^	MMA ^1^	B4 ^1^	MB4 ^1^	HM10% ^1^
MUM 23.14	−	−	−	+	−	+
MUM 23.15	−	−	−	+	−	+

+—activity detected; −—activity absent. ^1^ CREA: Creatine Sucrose Agar; CGA: Calcium Carbonate Glucose Agar; MMA: Modified Malt Extract Agar (with calcium carbonate); B4 (with calcium acetate); MB4: Modified B4 (with calcium carbonate); HM10%: DSMZ 372-Halobacteria medium supplemented with 10% NaCl (*w*/*v*).

## Data Availability

All relevant data are presented in the paper. The nucleotide sequences were deposited in the GenBank database under the accession numbers OR081765-OR081766 (LSU), OR081767-OR081768 (ITS), and OR074926-OR074926 (*rpb2*). The phylogenetic data can be accessed on Figshare via the following link: https://figshare.com/s/c68a893bc9b8f9f3c5d7 (accessed on 21 June 2023).

## Supplementary Materials

The following supporting information can be downloaded at https://www.mdpi.com/article/10.3390/jof9090916/s1, Figure S1: Visual representation outlining the measurement process in the temperature, NaCl, and pH assays. The pre-segmentation of the culture plate serves to establish a reliable reference line for measurements. This ensures that, particularly when colonies exhibit irregular shapes, diameter measurements remain impartial, mitigating any potential bias towards measuring the larger diameter, for instance.
