# Supplementary material for: Expanding the Microcolonial Black Fungi Aeminiaceae Family: Saxispiralis lemnorum gen. et sp. nov. (Mycosphaerellales), Isolated from Deteriorated Limestone in the Lemos Pantheon, Portugal
Source: J Fungi (Basel). 2023 Sep 10;9(9):916. doi: 10.3390/jof9090916 (PMC10533162; doi:10.3390/jof9090916)
Supplement: Supplementary file 1 [file jof-09-00916-s001.zip › jof-2492676-supplementary.pdf]

Supplementary Materials

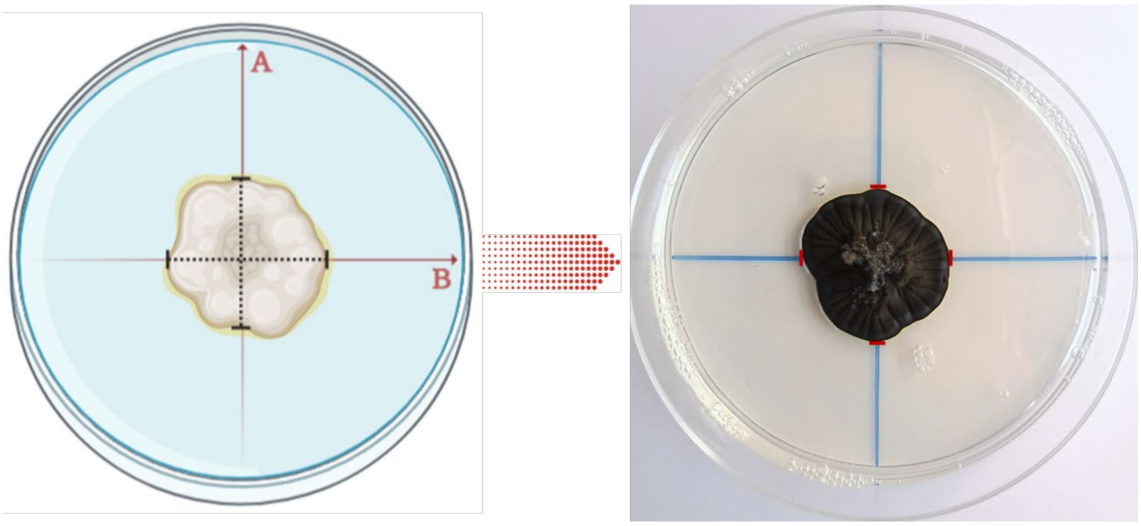

Figure S1. Visual representation outlining the measurement process in the temperature, NaCl and pH assays. The pre-segmentation of the culture plate serves to establish a reliable reference line for measurements. This ensures that, particularly when colonies exhibit irregular shapes, diameter measurements remain impartial, mitigating any potential bias towards measuring the larger diameter, for instance.
